# A Deep Autoencoder-Based Convolution Neural Network Framework for Bearing Fault Classification in Induction Motors

**DOI:** 10.3390/s21248453

**Published:** 2021-12-18

**Authors:** Rafia Nishat Toma, Farzin Piltan, Jong-Myon Kim

**Affiliations:** Department of Electrical, Electronics and Computer Engineering, University of Ulsan, Ulsan 44610, Korea; rafiatoma.eceku@gmail.com (R.N.T.); piltanfarzin@gmail.com (F.P.)

**Keywords:** bearing fault diagnosis, condition monitoring, convolution neural network (CNN), deep autoencoder (DAE), motor current signal, residual signal

## Abstract

Fault diagnosis and classification for machines are integral to condition monitoring in the industrial sector. However, in recent times, as sensor technology and artificial intelligence have developed, data-driven fault diagnosis and classification have been more widely investigated. The data-driven approach requires good-quality features to attain good fault classification accuracy, yet domain expertise and a fair amount of labeled data are important for better features. This paper proposes a deep auto-encoder (DAE) and convolutional neural network (CNN)-based bearing fault classification model using motor current signals of an induction motor (IM). Motor current signals can be easily and non-invasively collected from the motor. However, the current signal collected from industrial sources is highly contaminated with noise; feature calculation thus becomes very challenging. The DAE is utilized for estimating the nonlinear function of the system with the normal state data, and later, the residual signal is obtained. The subsequent CNN model then successfully classified the types of faults from the residual signals. Our proposed semi-supervised approach achieved very high classification accuracy (more than 99%). The inclusion of DAE was found to not only improve the accuracy significantly but also to be potentially useful when the amount of labeled data is small. The experimental outcomes are compared with some existing works on the same dataset, and the performance of this proposed combined approach is found to be comparable with them. In terms of the classification accuracy and other evaluation parameters, the overall method can be considered as an effective approach for bearing fault classification using the motor current signal.

## 1. Introduction

Rotating machinery is among the most pervasive and substantial components of the industrial sector. Whether the system is mechanical or electro-mechanical, one or more rotating machines are involved; examples include motors, generators, turbines, gearboxes, drive trains, automobile, and aircraft engines. Due to rapid industrialization and automation, the use of complex rotating machinery has increased by a lot, which increases the chance of multiple and significant faults occurring because of a generating fault in any single component [1]. Among all the various types of rotating machinery, induction motors (IMs) are the most commonly used because of their vigorous design, high productivity, reliability, and low cost [2]. In general, the IM needs to operate uninterrupted over a long time and under difficult operating scenarios. The operating conditions and unfavorable environment in many cases initiate different faults and may eventually lead to undesirable downtime, huge economic losses, and in the worse case, human causalities [3]. To avoid these unwanted situations, the fault diagnosis mechanism has emerged as an important part of the prognosis and health management (PHM) techniques. Research on the fault diagnosis of rotating machinery recently became a very popular topic, and many significant breakthroughs were achieved because of the speedy development of artificial intelligence. Designing a robust and accurate condition monitoring system can improve the fault diagnosis system by reducing maintenance costs, as well as by increasing reliability, productivity, and safety. It becomes very challenging to properly carry out this type of research work in practical industrial circumstances because of the complex, changing, and noisy environments that exist around rotating machinery, which makes it almost impossible to collect noise-free and accurate signals with proper fault information [4]. Statistical reports reveal that among different parts in IM, faults occurring in the bearing are a common phenomenon. Specifically, for small and large size machines, the rate of bearing faults occurring is approximately 90% and 40%, respectively [5]. Bearing faults can be initiated at the time of manufacture or during the period of operation.

Signals recorded from an IM may contain fault-specific information. In general, an impulse is initiated in the bearing fault signal when the bearing collides with the defective contact surface, and due to the damped oscillation, it generates a strident transient response. This recurrent transient response holds the necessary information about the bearing condition [6]. If an accurate analysis of the transient response can be performed, then any possibility of a fault occurring in bearing elements in the future can be identified at an early stage, which may avoid unpredicted downtime as well as make the industrial operation more efficient by avoiding huge monetary loss.

In general, the model-based [7,8] and data-driven-based [9,10] techniques are considered as the basic fault diagnosis mechanisms. Model-based fault diagnosis identifies the faults by using a small dataset, but it needs to model the system’s dynamics accurately. In highly nonlinear and uncertain conditions situations, it is difficult or impossible. Additionally, the technology of recent times ensures the availability of sensors for collecting various types of signals from machines to be used in the various fault monitoring systems. The availability of abundant data makes the data- driven -based approach in the fault diagnosis field very popular. The basic steps of the data-driven-based method are the acquisition of multiple state signals, extracting and selecting important features carrying fault signatures, and finally, classifying faults with machine learning algorithms [11,12,13]. Among the steps, the feature selection and extraction process is laborious and time-consuming; not only does it require deep knowledge of advanced signal processing techniques but also a great understanding of the working process and fault signals of the IM system [14]. Generally, vibration [15,16], current [17], acoustic emission [18,19], electromagnetic signals [20], and thermal imaging [21] are the signal types applied to diagnose faults. Therefore, it is very important to investigate and build a relationship between the recorded signals and corresponding types of bearing defects.

The fault signatures initiated in the bearings of IM mainly depend on fault-specific harmonic frequencies generated due to inner race, outer race, cage, or rolling element faults, the rotational speed of the rotor, or the geometrical dimensions of the bearing. Single point fault localization and diagnosis of fault in the entire bearing can be investigated with the additional installation of vibration sensors (accelerometer) [22]. The installed sensor can be difficult to access when located in a remote place, contributing to the overall costliness of the fault diagnosis process. To deal with these difficulties, fault diagnosis with motor current signal analysis (MCSA) has been proposed as an alternative way by many researchers as it does not require any additional sensors mounted around the bearing, which makes the data acquisition system non-invasive in nature and very cost-effective. Martínez-Montes et al. applied MCSA to measure the fault severities of two different bearing elements, the cage and the rolling elements, in a 5.5 kW IM [23]. Along with the bearing faults, eccentricity and broken rotor bar fault detection were also investigated by estimating the fault-related frequency and applying a matched subspace detector [24]. Techniques such as wavelet transform and short-time Fourier transform were also studied in [25,26,27] for fault detection from a stator current signal. Multiple signature analysis, such as the combination of MCSA and stray flux analysis, was introduced in [28], and it was found to detect mechanical faults in IM with good precision. Furthermore, the inner race faults were diagnosed by current, vibration, and stray flux signal in [29], where a 4 kW IM was involved in the experiment. From the comparative analysis, the researchers concluded that in the case of a mechanical fault, MCSA is more sensitive than stray flux, whereas in the case of misalignment and eccentricity, the stray flux becomes more sensitive.

Fault diagnosis from raw sensor data becomes computationally inefficient since the size of the original signal is generally large and the whole signal stream might not be adequate for fault identification. To reduce dimensionality, different signal processing techniques are carried out to extract useful features in different domains such as the time domain, frequency domain, and time–frequency domain. Later, the extracted features are fed as the input of various machine learning (ML) and deep learning (DL) methods for fault diagnosis. Machine learning approaches used for bearing fault classification include support vector machine (SVM), K-nearest neighbors (KNN), gradient boosting decision tree (GBDT), random forest (RF), principle component analysis, and artificial neural network [30,31,32,33]. The mentioned approaches require a fair amount of historical fault signature data (online dataset or laboratory measurement data) to train the model.

With the availability of high-volume data, the performance of DL approaches has improved. DL approaches have been successfully implemented in various research areas such as speech recognition, object detection, image classification, and fault diagnosis [34]. An overview of the amazing performance of CNN in fault diagnosis can be found in [35]. The main difference between classical ML and DL methods is that the accuracy of the classical ML model largely depends on the appropriate feature extraction and selection approach, whereas the DL inherits the automatic feature extraction ability. Although the combination of the signal processing techniques and classical ML/DL algorithms can also extract valid features and classify faults very efficiently, the performance largely depends on the input of a suitable amount of training samples.

In the industrial environment, it is quite difficult to collect a huge amount of noiseless data or data containing low noise with correct labeling. Other less forthright issues exist, such as when the results obtained from training and testing samples do not exhibit the same distribution, when there is a deficit in the amount of training data, and when samples are contaminated with different types of noise at different time instances of the recording [36]. Generally, in the real industrial sector, the collected bearing data are not labeled during acquisition [37]. The process of labeling is performed afterward when proper labeling cannot be confirmed. The lack of accurately labeled data will create challenges for the mostly applied supervised learning methods in the field of fault diagnosis. Furthermore, the complexity among different conditions of data, the time delay of acquiring raw signals, and imbalances occurring among different fault samples are some other technical challenges of the supervised fault diagnosis process [38].

For the challenges discussed above, sometimes it is beneficial to use unsupervised learning approaches. The autoencoder (AE) is considered as one of the promising unsupervised methods that can effectively learn features from unlabeled data. The AE approach is also being applied as a prominent dimensionality reduction mechanism in the rotating machinery fault diagnosis system [39,40] due to its high efficiency and ease of implementation. In fault diagnosis problems, the AE model is trained with the normal state data only, and it learns discriminative features that can be considered as the feature extraction mechanism for estimating the system state. There exist several extensions of conventional AE. Among them, the denoising AEs help to learn features that make differences among states in the highly noisy system [40]. Another form of AE named sparse autoencoder (SAE) is also implemented effectively in fault diagnosis systems by many researchers [14,41,42]. A combination of the SAE-deep belief network (DBN) is applied to fuse multi-sensor features for diagnosis bearing faults [43]. The variational AE (VAE) method was also applied in combination with deep generative models [44] and by using separate latent variables for each health state [45] for bearing fault diagnosis. In addition, the fault mode identification is performed by Huang et al. by combining recurrent neural network (RNN)-based VAE where the model preserves high-dimensional data information [46]. In [47], a convolution sparse autoencoder was designed for image recognition, where the convolutional autoencoder (CAE) was utilized for specifying the feature maps of the input. The ability to achieve good performance of AE with a small amount of data makes it a reasonable alternative to CNN as it requires huge data to perform. Many researchers have combined the AE and CNN in different fields such as fault diagnosis and image classification with a limited amount of data [40,48,49].

However, the CNN was designed to deal with the 2-D image of a large sample size. The one-dimensional CNN (1-D CNN) has been developed in recent times to deal with one-dimensional signals and has achieved good performance in terms of accuracy and computational time [50]. Researchers applied the 1-D CNN in real-time fault diagnosis and it not only achieved high performance but also can eliminate the manual feature extraction phase [50,51,52,53].

In this paper, a novel bearing fault diagnosis approach has been presented based on deep autoencoder (DAE) and 1-D CNN to address the drawbacks mentioned for supervised algorithms using motor current signal analysis (MCSA). Here, the DAE technique is used to substitute the traditional two-state control approach for approximating the behavior of the signal. In the beginning, with the normal state of MCSA, the DAE is trained and taught the latent coding, which denotes the equivalent nonlinear function of the bearing considering only the normal operating state. After model training with the normal state data, the current signal with the unknown condition is provided as the input of the DAE model, and with the learned latent coding from the normal data, the estimation of the new unknown signal state is performed. After that, the mean squared error (MSE) of the signal, also known as the residual signal, is calculated with the difference between the real and estimated signals of the data from the unknown state by the DAE model. The generated residual signals from DAE of different conditions act as an indication of dissimilarities of the different faulty signals, and these discriminative features help to improve the accuracy of the fault classification approach. At last, a 1-D CNN is designed with the residual signals as input to classify multiple types of faults of the bearing.

The major contributions of this work can be summarized as follows.

1. A novel data-driven approach based on DAE and 1-D CNN is presented using MCSA to investigate multiple fault states in the bearing of an induction motor.
2. An unsupervised DAE-based approach is introduced for the initial identification of faulty and normal state current signals for induction motors.
3. The fault diagnosis model is evaluated through a publicly available current signal dataset, and the final findings are compared with some previous works on the same dataset.

The rest of the paper is organized as follows. The experimental setup and data collection details are provided in Section 2. The details of the data segmentation and overall structure of the proposed model are demonstrated in Section 3. The experimental results of the proposed model and comparison with existing works on the same dataset are provided in Section 4. Finally, Section 5 includes the conclusion.

## 2. Experimental Setup and Data Acquisition

**Figure 1:**
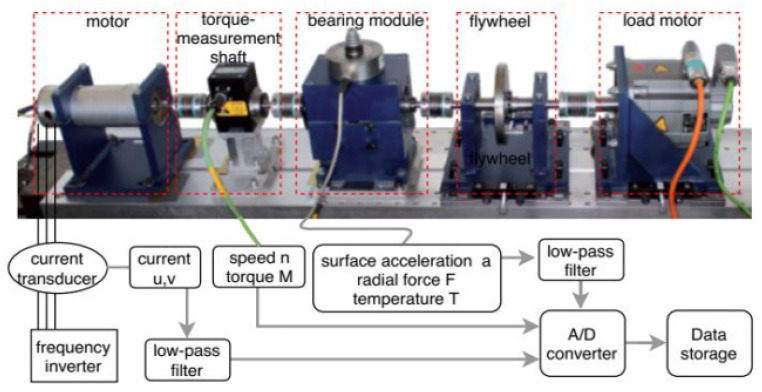
Schematic diagram of the test rig.

The current signal of IM used in this work was obtained from the Kat-Data Center contributed by the Mechanical Engineering research center of Paderborn University, Germany [54]. In addition to the current signal, this dataset also contains vibration signal, temperature, torque, speed, and radial load measurements of different operating conditions of the IM. The test rig was composed of an IM, torque-measurement shaft, a module of bearing, a flywheel, and finally, a load motor (Figure 1). Here, a frequency inverter was used to operate the 425 W permanent magnet synchronous motor (PMSM) with a switching frequency of 16 kHz. The model used for the PMSM and the frequency inverter are Type SD4CDu8S-009, Hanning Elektro-Werke GmbH and Co. KG, and KEB Combivert 07F5E 1D-2B0A, respectively [54].

In the data acquisition phase, a total of 32 different experimental bearings were involved, among them, 6 were normal bearings, 12 damaged bearings where damage was induced in an artificial manner, and another 14 faulty bearings with accelerating lifetime tests. The artificial damage in the bearing is created using three methods: drilling, manual electric engraving with a damage length of 1–4 mm, and electric discharge machining. Here, the appropriate directions of geometrical sizes of the cracks in bearings were assigned according to the VD1 3832 (2013) standard. For the accelerating lifetime test, plastic deformation damage, damage by pitting, and fatigue damage techniques were applied for the inner race and outer race faults. In case of injecting faults in bearings, the fault measurements (the bearing geometry, location of fault, size of damage) followed the 15,243 (2010) standards, which makes the overall data acquisition process more reliable. Additionally, to make the overall data acquisition process robust and acceptable, different faults with a wide range of severity levels were tested several times with various operating conditions as given in Table 1.

With a current transducer of model LEM CKSR 15-NP, the current signal for two different phases was measured for each of the operating conditions mentioned above. Among the 32 different signals available in the dataset, data from 17 bearings with 3 different conditions were considered in this analysis. There are 20 measurements for each of the bearings listed in Table 2, where each instance holds 4 s of recording. The signal is passed through a 25 kHz low pass filter and then sampled at a rate of 64 kHz. For our analysis, we divided 4 s of data into segments of 1 s, which makes the data dimension for our final analysis 1320 ∗ 64,000 for three different classes, named normal (class 0), outer race fault (class 1), and inner race fault (class 2).

## 3. Materials and Methods

**Figure 2:**
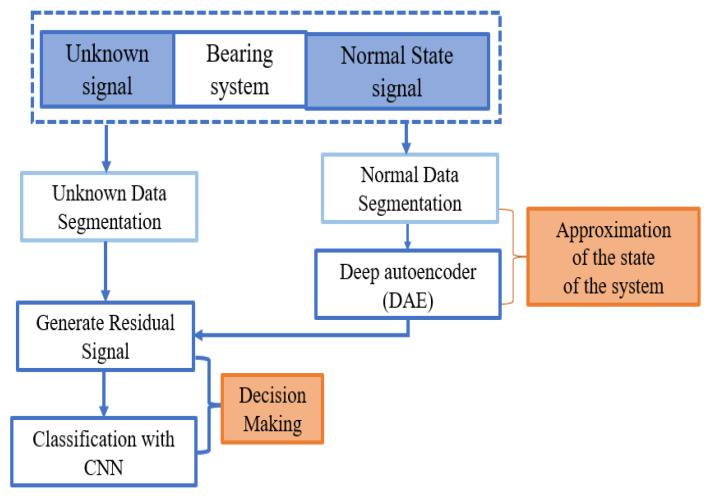
The designed framework of a DAE-CNN-based fault classification model.

A framework of the overall methodology is presented in Figure 2 for classifying three different conditions of bearing using the current signal of IM. In the beginning, a deep autoencoder (DAE) is trained only with the normal state-bearing data to generate a nonlinear function approximation of a system. After that, the residual signal is generated by the difference between the original current signal and the estimated current signal generated by the DAE using the learned nonlinear approximation with the normal state of the system. In this case, the anomaly detection mechanism of the autoencoder is applied to identify the deviations of the faulty signals from the original signal by generating a residual signal. At the last stage, the discriminative nature of the residual signal due to different bearing conditions is considered as the representation of the individual bearing states of the system and applied as the input of the CNN to classify three different conditions of the bearing, including one normal and two faulty in IM.

### 3.1. Bearing Fault Frequencies

**Figure 3:**
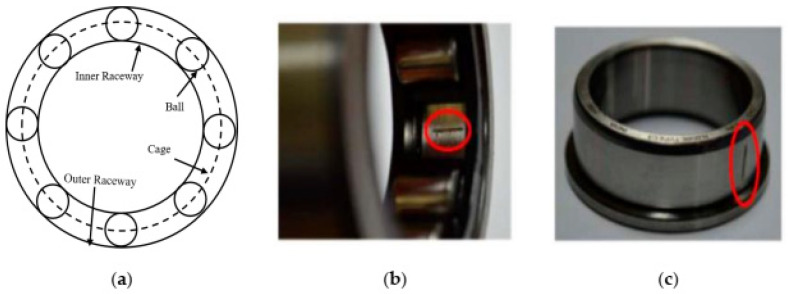
(**a**) Geometric structure of bearing; (**b**) fault in outer raceway; (**c**) fault in the inner raceway.

The rolling element bearings (REB) are generally used to make the rotor operation smooth by reducing the friction. They also have to operate for a long time under heavy load conditions. Because of this, the bearing fault is the most frequently occurring fault in IMs and requires thorough monitoring to avoid damage that can hamper the whole industrial operation. The REB contains four basic elements, the inner race, outer race, cage, and rolling elements. In bearings, the outer ring and inner ring are mounted on a rotating shaft, whereas the rolling elements are placed in a closed cage having the same distance from one another. Different types of faults such as pitting or flaking can be generated in these elements due to adverse operating conditions, such as improper installation and lubrication, material fatigue, and contamination in lubricating materials. Single element faults such as those of the outer race, inner race, or roller faults occur most often, but multiple faults can also be generated simultaneously in various elements. We consider the motor current signal for two faulty conditions (Figure 3) along with the normal bearing condition in this work.

When any fault generates during operation and the roller passes across the defect point in every rotation, a shock impulse is creating having a characteristic defect frequency. The damage frequencies of different elements can be calculated with the geometric parameters of the bearing and the rotational speed along with the help of the Equations provided as (1)–(4):

(1) $$\mathrm{Inner}\;\mathrm{race}\;\mathrm{fault}\;\mathrm{frequency}:\;F_{inner}=\frac{N_{ball}}{2}\times f_{m}\times \left(1+\left(\frac{D_{ball}}{D_{cage}}\times \cos\beta \right)\right)$$

(2) $$\mathrm{Outer}\;\mathrm{race}\;\mathrm{fault}\;\mathrm{frequency}:\;F_{outer}=\frac{N_{ball}}{2}\times f_{m}\times \left(1-\left(\frac{D_{ball}}{D_{cage}}\times \cos\beta \right)\right)$$

(3) $$\mathrm{Roller}\;\mathrm{fault}\;\mathrm{frequency}:\;F_{roller}=\frac{D_{cage}}{2D_{ball}}\times f_{m}\times \left(1-{\left(\frac{D_{ball}}{D_{cage}}\times \cos\beta \right)}^{2}\right)$$

(4) $$\mathrm{Cage}\;\mathrm{fault}\;\mathrm{frequency}:\;F_{cage}=\frac{f_{m}}{2}\left(1-\left(\frac{D_{ball}}{D_{cage}}\times \cos\beta \right)\right)$$

Here, $N_{ball}$ is the number of rolling elements (balls), $D_{ball}$ is the ball diameter, $D_{cage}$ is the cage diameter, β is the angle measurement of the balls, and $f_{m}$ represents the rotational frequency.

When the bearing fault occurs, a radial displacement is created between the stator and rotor, and oscillations, as well as fault frequencies, are generated in the current signals because of the radial motion. Later, the load torque and the rotating eccentricity develop some fluctuations that result in variations in the values of inductance and cause amplitude, frequency, and phase modulation. The current equation due to the occurrence of bearing faults can be expressed as:

(5) $$i\left(t\right)=\overset{\infty }{\underset{k=1}{\sum }}i_{k}cos(\omega_{C_{k}}t+\phi )$$

where ϕ is the phase angle, and $\omega_{C_{k}}$ represents the angular velocity, and

(6) $$\omega_{C_{k}}=\frac{2\pi f_{bearing}}{p}$$

Here, p is the pole pair number of the operating machine, and $f_{bearing}$ indicates the harmonic frequency of the current signal and can be written as, $f_{bearing}=\left|f_{s}\pm mf_{v}\right|$. Here, $f_{s}$ and m denote the supply frequency and the harmonic index, respectively. However, $f_{v}$ can be either $f_{inner}$ or $f_{outer}$. Therefore, by applying the frequency auto search algorithm, the approximation of fault frequencies can be possible [55]. In some cases, the harmonics generated due to the bearing fault and the noise frequencies become almost similar, which later creates a problem in differentiating the actual fault frequencies [56].

**Figure 4:**
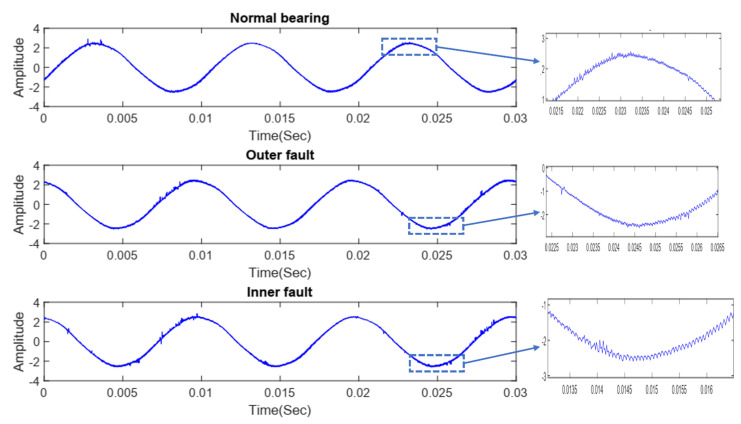
Time−domain signal of the different bearing conditions.

The representation of three different conditions of the current signals at the time domain is given in Figure 4, where all the signals exhibit subtle differences if we consider the zoom view.

**Figure 5:**
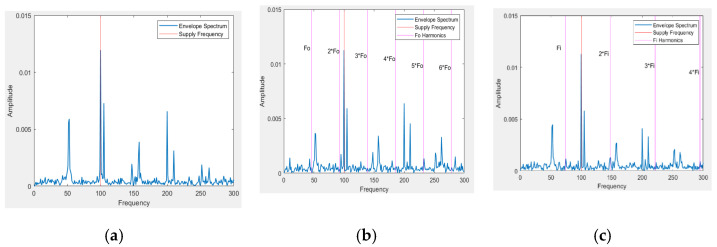
Envelope spectrum analysis of three conditions of current signals: (**a**) normal, (**b**) outer fault, and (**c**) inner fault.

The envelope analysis is considered effective in analyzing the fault frequencies of different bearing fault conditions [57]. In Figure 5, the envelope spectrums of three different conditions are presented to exhibit the supply and corresponding fault frequencies. Here, the supply frequency (100 Hz) is visible for all conditions. However, for the inner and outer fault conditions, the envelope spectra of the current signal do not show a peak at the inner and outer frequency harmonics for all instances. The absence of indication of faults in the current signal is because of the damped signal condition, presence of noise as well as multiple disturbances, and the indirect transmission of the fault signatures in the drive train through torque variations. Therefore, the features extraction from the current signal becomes difficult and challenging, which makes essential the development of efficient feature learning approaches for diagnosis bearing faults with a current signal [58].

### 3.2. Data Segmentation

In an industrial setup, the collection of a large-scale dataset with proper labeling is time-consuming, laborious, and makes the overall system design too complex. However, to implement deep learning-based methods, high dimensional training data improves the learning model to learn efficiently. In addition, in the case of implementing a 1-D signal in a convolution neural network approach, the input data dimension will influence the overall architecture of the model. As the input shape increases, the number of input nodes and hidden layers also increases. Such a large and deep structure may provide good performance, but it also requires a large amount of time to learn by the model and there is the possibility of overfitting. To resolve this issue and convert the data meaningfully, a resampling mechanism is applied on different states of the current signal before the autoencoder, which prepares a sequence of frames. Here, each frame contains the same number of data points collected at the time of each revolution period. Three steps, provided below, are followed for the data segmentation before using data as the input of the autoencoder.

1. Determining the number of rotations by the bearing in one second as the number of revolutions accomplished in one second (*RPS*) can be estimated with the formula stated below:
  (7) $$Rotations\;per\;second\left(RPS\right)=\frac{rotating\;speed\;in\;rpm}{60}$$
2. Determining the time required for one complete rotation as
  (8) $$TOR=\frac{1}{RPS}\;$$
3. Finally, the total number of data points recorded during one revolution can be found by Equation (9).
  (9) $$F_{frame\_size}=f_{sampling}\times TOR$$

Here,$f_{sampling}$ and $F_{frame\_size}$ represent, respectively, the sampling frequency and each frame length of the resampled signal in terms of numbers of data points.

Thus, when the speed of the bearing rotation is 1500 rpm, the parameters in Equations (7)–(9) are calculated as *RPS =* 15, *TOR =* 0.04, and $F_{frame\_size}$ = 2560 for 1-s data.

### 3.3. Deep Autoencoder (DAE)

**Figure 6:**
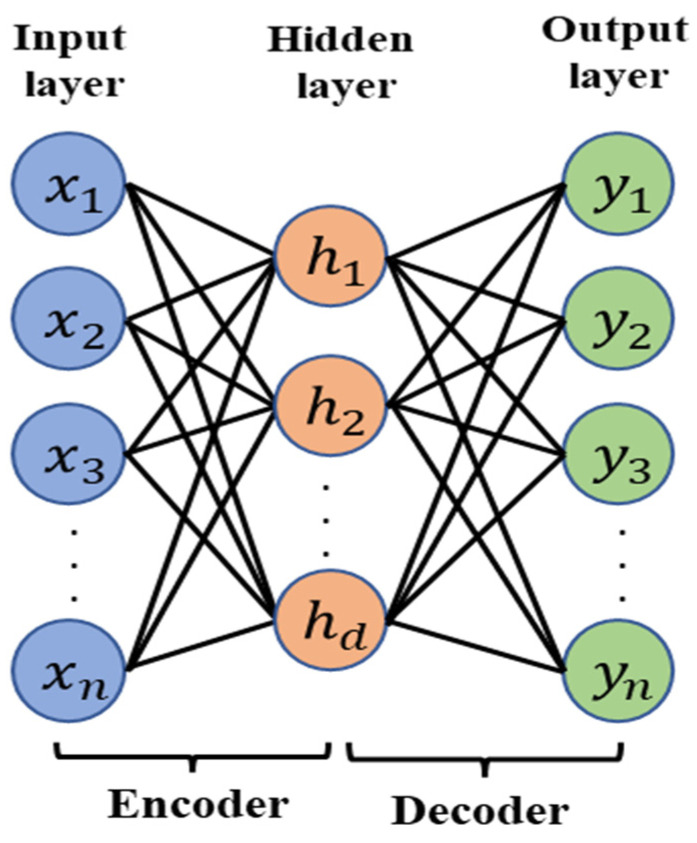
Basic architecture of the autoencoder.

The autoencoder based on a deep neural network is considered as one of the most robust unsupervised learning models of the last few decades. With the unsupervised model, it becomes possible to extract effective and discriminative features from a huge unlabeled data set, which makes this approach widely applicable for the extraction of features and dimensionality reduction [36]. Basically, an autoencoder consists of a fully connected three-layer neural network where the encoder contains input and hidden layers and the decoder part comprises hidden and output layers. The encoder transfers the input data with a higher dimension into a feature vector with a lower dimension. After that, the decoder converts the data back to the input dimension. One of the main priorities of the deep neural network is to build a complex nonlinear relationship among the input data, which also helps in the autoencoder to effectively reconstruct the output of the decoder. Therefore, the reconstruction error will be decreased simultaneously through the overall training period and significant features will be stored in the hidden layer. Finally, the hidden layer output will depict the efficiency of the feature extraction of the designed autoencoder. Figure 6 represents the configuration of the basic autoencoder.

For the n-dimension input data samples, $X=\left[x_{1},x_{2},\ldots ,x_{n}\right]$, the output/activation of the hidden layer h with *m*-dimension (*m* < *n*) can be calculated as Equation (10):

(10) $$h=f^{h}\left(W^{\left(1\right)}x+b^{\left(1\right)}\right)$$

Here, $W^{\left(1\right)}x,b^{\left(1\right)}$ and $f^{h}$ represent the weight matrix connecting the input and hidden layer, bias, and activation function, respectively.

After the decoding process, the reconstructed signal, $\tilde{x}$ at the output layer can be expressed as:

(11) $$\tilde{x}=f^{o}\left(W^{\left(2\right)}h+b^{\left(2\right)}\right)$$

Here, $W^{\left(2\right)},b^{\left(2\right)}$ represent the weight matrix and the bias vector of the output layer. The activation function used for both encoder and decoder parts is generally set as a sigmoid function, $f\left(t\right)=1/\left(1+e^{-t}\right)$ or any other activation function depending on the data type. The training process begins with some initial values of weights and biases.

During the training process, the parameters need to adjust for minimizing the reconstruction error between the original input data and the reconstructed output. The reconstruction error is quantified by the mean squared error (MSE), as mentioned in Equation (12), which is applied in our analysis.

(12) $$L\left(W,b\right)=\frac{1}{N}\overset{N}{\underset{n=1}{\sum }}{\left(x-\tilde{x}\right)}^{2}$$

In other cases, if the input values exist between 0 and 1, binary cross-entropy loss will be calculated as the reconstruction error with Equation (13):

(13) $$L\left(W,b\right)=-\frac{1}{mn}\overset{m}{\underset{i=1}{\sum }}\overset{n}{\underset{j=1}{\sum }}\left[x_{j}^{\left(i\right)}log{\tilde{x}}_{j}^{\left(i\right)}+\left(1-x_{j}^{\left(i\right)}\right)logx_{j}^{\left(i\right)}\right]$$

In this analysis, a deep autoencoder (one which uses more than one hidden layer) is applied to find an approximation of the normal state of the bearing, whose architecture is provided in Table 3.

The scaled exponential linear unit (SELU) is applied as the activation function for both hidden and output layers of the DAE in this analysis. The recorded current signal has both positive and negative values; since SELU is a non-saturating type of activation function, it is a good choice for the type of signal used here, and it also tackles the vanishing gradient problem that occurred in the deep network architecture. Additionally, the normalization properties of SELU help to make the training process fast by converging the deep neural network quickly. The SELU can be defined as Equation (14):

(14) $$f_{SELU}=\lambda \left\{\begin{array}{cc} \alpha e^{x}-\alpha ; & x\leq 0\\ x; & x>0 \end{array}\right.$$

Here, the coefficient values for λ and α are set to approximately 1.05 and 1.6731, respectively, according to [59]. In this work, the optimizer used for updating the weight is adaptive moment estimation (Adam). This optimizer technique is becoming very well-known because of its ability to memorize prior gradients as well as prior squared gradients exponentially decaying average values of the loss function [60].

### 3.4. Generation of the Residual Signal

To generate the residual signal with the designed autoencoder to apply it as the input of the CNN is one of the most significant parts of this research. In the training phase, normal-state bearing data are fed to the DAE, and it learns the nonlinear behavior of the system. Once the training phase is over, current signals for different states of the bearing are provided as input to the DEA. In this case, all the signal instances are different from those that are used in the training phase. In response, the DAE provides an estimated signal for each instance of the input signal. Next, the difference between the original input signal and the estimated signal from DAE is calculated, which is known as the residual signal. The computation of the residual signal, $r_{\hat{x}}\left(n\right)$ can be calculated as [40]:

(15) $$r_{\hat{x}}\left(n\right)=x\left(n\right)-\hat{x}\left(n\right)$$

Here, $x\left(n\right)$ and $\hat{x}\left(n\right)$ represent the raw time domain motor current signal and reconstruction signal with the model developed with the normal state data, respectively.

Finally, we arranged the residual signal with three different conditions of motor current signal for normal bearing, outer race fault, and inner race fault and used it as the input of the 1D-CNN in the next step to perform fault classification.

The reconstruction error will be different for signals of different bearing states. The DAE model is trained with normal bearing state data, so when it estimates a normal signal instance, the reconstruction error will be small. However, when DAE estimates for a current signal that contains a fault signature, there is a high possibility that the reconstruction error will deviate significantly from the previous case. Generally, various types of faulty condition signals contain different characteristics and amplitude levels, and the relative statistical parameters also vary according to the signal amplitude. As a result, the difference between the time domain faulty state signals and the estimated signal by the DAE (residual signal) will also vary depending on which type of fault is present. For this reason, the computed residual signal can be used as discriminative features not only to detect the present condition of any system but also to diagnose fault classification performance of IM bearing.

### 3.5. Convolution Neural Network (CNN)

A convolution neural network (CNN) is a deep learning-based supervised algorithm that combines feature extraction and feature classification approaches. In general, the CNN is a feed-forward, deep network having full connectivity through the adjoining layers and one that performs better in comparison with other general supervised techniques. The ability to automatically learn high-dimensional features and solve the overfitting problem in ML approaches makes CNN a very effective technique in large-scale applications. The CNN is built with an input layer, multiple convolution layers, pooling layers, a fully connected layer, and an output layer. Each layer performs distinct roles, which are performed automatically within the architecture [61]. Additional inclusion of optimization parameters, dropout layers, and batch normalization, help CNN to decrease its dependency on the training data [62].

The original time-series data or the images of interest are passed to the convolution layer from the input layer. The heaviest computational task occurs in the convolution layer when a set of feature maps is generated. In each convolutional layer, a kernel having a local receptive field is used to perform convolution operations with the input data. After that, a bias term is added and the result passes through a nonlinear activation function, such as rectified linear unit (ReLU), to generate the output feature map, which acts as the input for the subsequent convolutional layer. ReLU is the most used function due to its ability to make the nonlinearity of CNN quite high. The convolution operation can be defined as Equation (16):

(16) $$X_{j}^{l}=ReLU\left(\underset{i\in M_{j}}{\sum }X_{i}^{l-1}\times \omega_{ij}^{l}+b_{j}^{l}\right)$$

Here, the ReLU operation can be calculated as Equation (17),

(17) $$x_{i}^{j}=max(0,x_{i}^{j^{\prime }})$$

Here, $X_{j}^{l}$ and $X_{i}^{l-1}$ represent the output and input layers of the convolution layer with *i-th* input feature map and *j-th* output feature map. Furthermore, *l* indicates the layer number, $\omega_{ij}^{l}$ represents the weight matrix, $b_{j}^{l}$ is the bias matrix, and ReLU represents the activation function.

After the convolution layer, a down-sampling layer is added to merge similar types of features, which reduces the size of the feature map and reduces the computation time by maintaining the same invariance in the characteristic scale. Therefore, the pooling layer reduces the data dimension without updating the weights of the parameters. It is important to set the stride parameter carefully as it plays an important role in this layer to reduce the resolution and preserve the numerical information. Max pooling, average pooling, norm pooling, logarithmic pooling, and stochastic pooling are different types of pooling approaches used in CNN. The output of the pooling layer for the *j-th* channel of the *t-length* feature can be expressed as:

(18) $$P_{j}\left(n\right)=\underset{0\leq n\leq \frac{t}{s}}{max}\left\{X_{j}(nW,\left(n+1\right)W\right\}$$

where $X_{j}$*, W, S* represent the input, width of the pooling window, and stride size, respectively.

After completing the multiple stacks of convolution and pooling layers, the outcome can be transferred to the final stage of CNN, named the fully connected layer. This layer utilizes the output from the last pooling layer to predict the classes of the provided data. Therefore, the input of this layer was generated by performing a weighted summation of a one-dimensional feature matrix expanded by all feature graphs. The output of this layer $y^{i}$ can be expressed as:

(19) $$y^{i}=f\left(w^{i}x^{i-1}+b^{i}\right)$$

Here, $x^{i-1}$, $w^{i}$, and $b^{i}$ are the feature vectors of one dimension, weight matrix, and bias, respectively. The output of the fully connected layer is a probability for each class or category in case of the classification problem. The probability is computed by a softmax activation function. The categorical cross-entropy loss function is used to calculate the gradients, which are to be utilized to update the weights in the training phase involving the loss function, which is expressed as follows:

(20) $$Loss\left(\theta \right)=-\frac{1}{n}\sum_{i=1}^{n}\sum_{k=1}^{K}y_{k}^{i}log({\hat{P}}_{K})$$

Here, $y_{k}^{i}$ and ${\hat{P}}_{K}$ represent, respectively, the target and estimated probabilities for *i-th* instances in the dataset containing the output class label *k*. The Adam optimization used for the autoencoder is also used for training the CNN.

**Figure 7:**
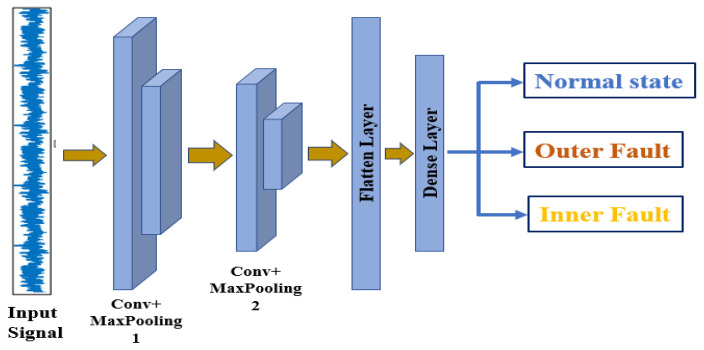
Architecture of the designed CNN.

The architecture of the CNN model applied in this work is shown in Figure 7, which connects the input layer with two convolution and max-pooling layers and finally one fully connected and three output layers.

The outline of the CNN model used in this fault classification task is presented in Table 4, where the details of layer type, output shape, and the total number of parameters are included.

### 3.6. Fault Classification Performance Evaluation Parameters

As our proposed approach classifies bearing faults from the motor current signal, we used commonly used evaluation parameters for classification problems such as precision, recall, F1-score, and accuracy. These parameters can be calculated using Equations (21)–(24).

(21) $$Precision=\frac{TP}{TP+FP}$$

(22) $$Recall=\frac{TP}{TP+FN}$$

(23) $$F1\_score=\frac{2\times Precision\times Recall}{Precision+Recall}$$

(24) $$Accuracy=\frac{TP+TN}{TP+FP+TN+FN}$$

## 4. Experimental Results and Discussion

A pipeline process consists of two steps: the first one performs the approximation of the nonlinear function, and the second stage represents the decision-making process for classification, and these are applied in the proposed method for fault classification. The nonlinear function approximation was performed through a DAE, which was trained with only the normal state data of the bearing and, by using the model, the residual signals were obtained for one normal state and two different faulty states.

**Figure 8:**
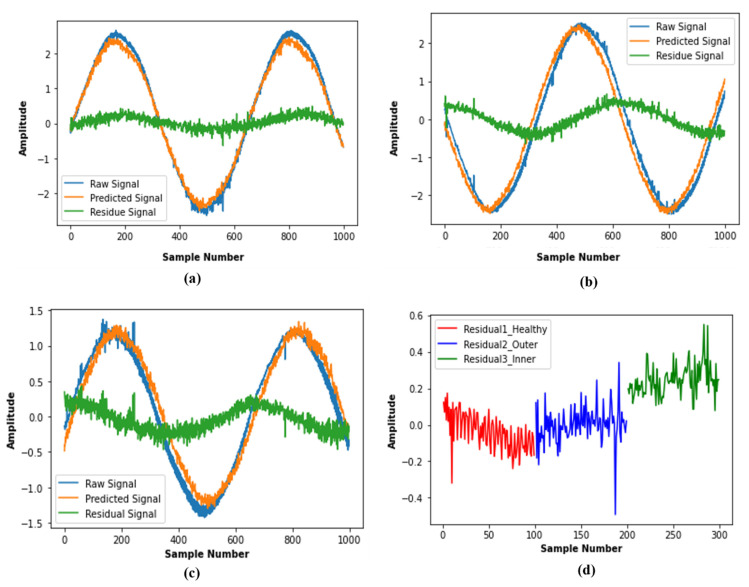
The raw, predicted, and residual signal of bearings corresponding to (**a**) normal, (**b**) outer race fault, (**c**) inner race fault conditions, and (**d**) residual values for 100 samples of three different conditions.

Initially, we started with one second of the current signal recording consisting of 64,000 samples, and after performing signal segmentation, we obtained 2560 samples for each frame. In the beginning, the normal condition segmented data samples of the motor current signal were only considered to train the DAE. Then, the same signal is again reconstructed with the trained model. From the difference of these two signals, the residual signal (also known as reconstruction error) was generated and used as the input of the CNN in the next step. In this training process of DAE, the segmented data are divided into training and test sets with an 80:20 ratio. Therefore, 2048 samples were used to train the DAE model, and the remaining 512 samples were used for testing purposes to calculate the validation error. After training the DAE model for 500 epochs, each 1-s data (64000 samples) segment of normal and faulty conditions was used to generate the residual signal. In the end, these residuals are applied as the input of the CNN, which helped the decision-making part in classifying the bearing conditions. Since the DAE model is trained initially with normal condition data, the residual signal generated due to normal state data is very low in magnitude with most values clustered around zero. Therefore, the small magnitude residual signal indicates a very small reconstruction error as the original data and the predicted data through DAE nearly resemble each other. Generally, when a fault occurred in a system, the faulty data showed some deviation from normal state data. Hence, when the residual signal is generated for signals of faulty condition, the amplitude of reconstruction error also becomes high as compared to the residual signal of the normal state data. We calculated the reconstruction error with mean square error and obtained the values 0.104, 0.386, 0.479 for normal, outer race fault, and inner race fault data, respectively. The raw signal, corresponding signal predicted by DAE, and residual signal for three different conditions are presented in Figure 8a–c. Additionally, 100 sample segments of residuals obtained for each of the signal types are plotted in Figure 8d to provide a clear visualization of the differences among the normal and faulty conditions in terms of the residual signal.

After obtaining the residual signals for all three conditions, 80% of these residual signal samples are used to train the subsequent CNN model, and the remaining 20% are used for the testing phase. While training the CNN model, 64 samples were grouped as data batches, and the training process is continued up to 500 epochs. We utilized a 2-layer CNN as mentioned in Section 3.5 and, by optimizing the model parameters, 99.6% accuracy was achieved on the test dataset.

To validate the performance of our proposed method, we compare the result with some other partially modified approaches. For the first one, we kept the CNN model unchanged and made the original segmented current signal as the input (Raw + CNN) to investigate whether or not the nonlinear signal approximation technique by DAE is playing a significant role in improving the model performance. The accuracy obtained by Raw + CNN was 61.06%, which is significantly lower than the proposed method. We also wanted to investigate whether the familiar machine learning algorithms could perform a good classification of the faults. For this purpose, a group of statistical features (SF) mentioned in Table 5 were extracted from the residual signal, and later, these features are used with support vector machine (SVM), random forest (RF), and k-nearest neighbor (KNN) individually. These three approaches are mentioned as RS + SF + SVM, RS + SF + RF, and RS + SF + KNN, respectively, in Table 6.

Finally, the evaluating parameters of all the mentioned approaches are listed in Table 6, and it is evident from the results that the proposed methodology with an accuracy score of 99.6% outperformed the other discussed methods.

**Figure 9:**
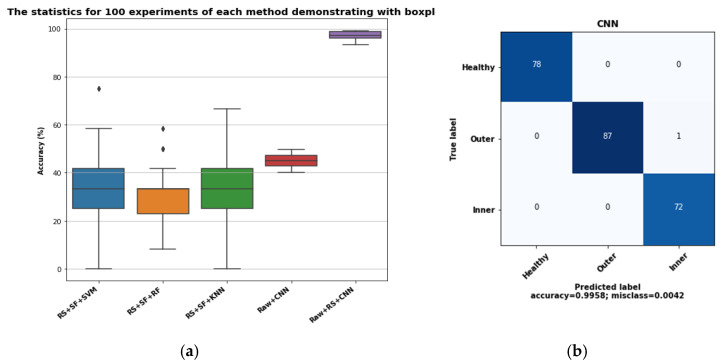
(**a**) The boxplot representing the accuracy matrix of over 100 experiments; (**b**) confusion matrix.

To observe the repeatability of our proposed model, all the experiments were executed 100 times and the resulting accuracy distribution was provided as box plots in Figure 9a. The confusion matrix of our proposed method is presented in Figure 9b, where only one sample is incorrectly classified with the designed CNN.

From the boxplot representation, the accuracy value of our proposed method did not significantly vary from the mean and median values throughout the experiments that confirm the repeatability of the outcome. Not only that, other approaches that involve only the CNN architecture also showed a small deviation in accuracy, and no outliers have been observed. However, the accuracy is a little higher than the ML classifiers but much lower than our proposed method. Therefore, the inclusion of an autoencoder-based approach with CNN helps to achieve high accuracy where signals have nonlinear characteristics. In general, the time domain current signal possesses non-stationary characteristics and the statistical properties of the data containing the same class may vary with time, which results in less discriminative features among the classes. For this reason, when it is directly set as the input of CNN, the model fails to assign proper weights at the training phase to accomplish good accuracy on the testing samples. On the other hand, the other three approaches involving characterization of the residual signal with statistical features show high deviation from the mean and median values, a large number of outlier samples, and results in a comparatively low accuracy in fault classification performance with the current signal.

Finally, we made a comparison with some other existing approaches where the same dataset is being used for the bearing fault classification. An information fusion (IF) technique was investigated by Hoang and Kang [58], and three different classifier algorithms were applied for fault classification with the motor current signal. In their approach, first they convert the current signal into a 2-D gray image and then apply a 2-D CNN to classify the images. Hence, they mentioned the CNN structure, where the number of convolution and pooling layers was four. However, in our analysis, we first apply an autoencoder and later use the generated reconstruction signal in 1-D CNN to classify the fault. Here, we used a two-layer 1-D CNN with two convolution and two pooling layers. In general, the computational complexity of the CNN model is proportional to the number of layers and the parameters. Due to the nature of residual signal of bearing states, high classification accuracy can be achieved with the help of a simple two-layer CNN. If we consider both model type and complexity, our model seems easy to implement and requires less time to accomplish. Furthermore, a combination of wavelet packet decomposition (WPD) and particle swarm optimization-based SVM was applied by [54] and achieved more than 85% accuracy in classifying the bearing fault with the current signal. Hsueh et al. applied empirical wavelet transform to transform the 1-D current signal into 2-D grayscale images and later to classify the fault with CNN [63]. Table 7 presents the comparison of the mentioned existing works with our proposed method.

From the comparisons among the experimental results and existing works, it can be concluded that the discussed data-driven-based method containing the DAE-CNN model can attain high accuracy in bearing fault classification with the motor current signal. In this two-step pipeline method, the DAE acts as an automatic and efficient feature learning approach, which also provides an approximation of the nonlinear behavior of the bearing system. The resultant residual signal enhances the fault diagnosis ability of CNN by providing discriminative features according to the bearing states. The overall method does not require any additional signal processing techniques for extracting features, which makes the approach less complex and less time-consuming. To make our method more reliable and robust, in our future analysis, we will consider changing the operating conditions of IM, including varying load and rotating speed. Therefore, the DAE-based CNN approach can be considered as an efficient and effective way to learn features and classify bearing faults by utilizing the motor current signal.

## 5. Conclusions

Due to the availability of sensor data, research has become more focused on data-driven based fault diagnosis techniques. Among the different sensor data available, analysis with the motor current signal data is considered a smart solution due to the advantages of low cost, easy access, and extensive technical support. In this analysis, a novel semi-supervised method is introduced to classify three different bearing fault states. The approach utilizes an unsupervised and supervised model simultaneously. In the beginning, the time-domain current signal is segmented considering the fundamental frequency, and then the deep autoencoder (DAE) is trained with the normal state data to estimate the function approximation of the system. After that, the residual signal is calculated from the difference between raw and estimated signals produced by the autoencoder for all conditions. In this step, the DAE helps to extract discriminative features from the current signal data without any labels. Lastly, with the residual signals, a two-layer CNN is constructed for identifying the bearing faults. The experiments were performed 100 times by randomly selecting the training/testing data set, and the result shows good stable convergence with high accuracy. This method does not require any previous knowledge about the system or any additional signal processing techniques for feature engineering. Furthermore, a comparison is presented with some reference approaches as well as some recent works to test the efficiency, which indicates that the DAE-based CNN method can be an efficient fault classification approach to classify different bearing faults. As proper data labeling is quite difficult in an industrial environment, this semi-supervised learning mechanism can be a promising alternative for supervised learning approaches in the fault diagnosis method. In our future work, we will try to improve the autoencoder model architecture to perform a better nonlinear model approximation to enhance the reliability and robustness of the system. In addition, a systematic hyperparameter tuning approach will be investigated for building an optimized CNN structure to make the decision-making approach more automated.

## Figures and Tables

**Table 1:** Operating conditions of the testbed.

Condition No	Rotational Speed (S) (rpm)	Radial Force (F) (N)	Load Torque (M) (Nm)
1	1500	1000	0.7
2	1500	1000	0.1
3	1500	400	0.7
4	900	1000	0.7

**Table 2:** Bearing data considered for analysis.

Type of Bearing	Bearing Code	Class Label
Normal Bearing	K001, K002, K003, K004, K005, K006	0
Outer Ring	KA04, KA15, KA16, KA22, KA30	1
Inner Ring	KI04, KI14, KI16, KI17, KI18, KI21	2

**Table 3:** The structure of the designed deep autoencoder (DAE).

Layer #	Layer Type	Number of Nodes	Activation
1	Input (Encoder)	2560	-
2	Hidden (Encoder)	1280	SELU
3	Hidden (Encoder)	640	SELU
4	Hidden (Encoder)	320	SELU
5	Hidden (Encoder)	128	SELU
6	Hidden (Encoder/Decoder)	32	SELU
7	Hidden (Decoder)	128	SELU
8	Hidden (Decoder)	320	SELU
9	Hidden (Decoder)	640	SELU
10	Hidden (Decoder)	1280	SELU
11	Output (Decoder)	2560	SELU

**Table 4:** Sequential model of a 2-layer CNN.

Layer (Type)	Output Shape	Number of Parameters
conv1d_1 (Conv1D)	None, 63,998, 64)	256
max_pooling1d_1 (MaxPooling)	(None, 31,999, 64)	0
conv1d_2 (Conv1D)	(None, 31,997, 32)	6176
max_pooling1d_2 (MaxPooling)	(None, 15,998, 32)	0
flatten_1 (Flatten)	(None, 511,936)	0
dense (Dense)	(None, 3)	1,535,811
Total params: 1,542,243		
Trainable params: 1,542,243		
Non-trainable params: 0		

**Table 5:** List of extracting statistical features from the residual signal.

Feature Name	Equation	Feature Name	Equation
RMS:	$X_{rms}=\frac{\sqrt{\sum_{i=1}^{N}x_{i}{}^{2}}}{N}$	Energy:	$E=\overset{N}{\underset{i=1}{\sum }}x_{i}{}^{2}$
Standard Deviation:	$S=\sqrt{\frac{\sum_{i=1}^{N}{\left(x_{i}-\mu \right)}^{2}}{N-1}}$	Kurtosis:	$x_{kurtosis}=\frac{1}{N}\frac{\sum_{i=1}^{N}{\left(x_{i}-\mu \right)}^{4}}{\sigma^{4}}$
Variance:	$S^{2}=\frac{\sum_{i=1}^{N}{\left(x_{i}-\mu \right)}^{2}}{N-1}$	Skewness:	$x_{skewness}=\frac{1}{N}\frac{\sum_{i=1}^{N}{\left(x_{i}-\mu \right)}^{3}}{\sigma^{3}}$
Crest factor:	$C_{f}=\frac{X_{max}}{X_{min}}$	Shannon-entropy	$H_{s}=-\overset{N}{\underset{i=1}{\sum }}x_{i}{}^{2}log(x_{i}{}^{2})$
Form factor	$F_{f}=\frac{\mu }{X_{rms}}$	Log-energy entropy	$H_{e}=-\overset{N}{\underset{i=1}{\sum }}log(x_{i}{}^{2})$

**Table 6:** The results of the evaluation parameters of five different approaches.

Methods	Evaluation Parameters			
	Recall	Precision	F1_Score	Accuracy (%)
RS + SF + SVM	0.42	0.41	0.41	41.67
RS + SF + RF	0.53	0.52	0.52	53.06
RS + SF + KNN	0.47	0.49	0.44	47.08
Raw + CNN	0.60	0.59	0.60	61.06
Proposed	0.99	0.99	0.99	99.6

**Table 7:** Comparison of accuracy metrics with existing works.

Applied Methods	Classification Accuracy (%)
IF + MLP [58]	98.3
SVM + IF [58]	98.0
KNN + IF [58]	97.7
WPD + SVM-PSO [54]	86.03
CNN + EWT [63]	97.3
Proposed (Raw + RS + CNN)	99.6

## Data Availability

The data are publicly available.
